# Cognitive Costs of Reappraisal Depend on Both Emotional Stimulus Intensity and Individual Differences in Habitual Reappraisal

**DOI:** 10.1371/journal.pone.0167253

**Published:** 2016-12-09

**Authors:** Catherine Nicole Marie Ortner, Mark Ste Marie, Daniela Corno

**Affiliations:** Thompson Rivers University, Kamloops, British Columbia, Canada; IRCCS Istituto Auxologico Italiano, ITALY

## Abstract

Recent models of emotion regulation suggest that the cognitive costs of reappraisal depend on stimulus intensity and habitual reappraisal. In the current experiment, we tested these hypotheses by manipulating the intensity of unpleasant and pleasant images, which participants reappraised, viewed, or suppressed their emotions to. To assess cognitive costs, we measured participants’ performance on a concurrent simple reaction time task. Participants also reported on their everyday use of reappraisal and suppression. Higher intensity stimuli were associated with greater cognitive costs of reappraisal, for unpleasant, but not pleasant pictures. Also, greater habitual reappraisal predicted lower cognitive costs of reappraisal and greater reductions in subjective feelings. Results support the role of stimulus intensity and habitual use of reappraisal in predicting the cognitive costs of reappraisal.

## Introduction

After being passed by an ambulance on the highway, you wonder if someone was in a serious car accident. However, you are close to a large town, so you tell yourself that in all likelihood the person will get to hospital on time and they will receive the medical attention they need. Any emerging worries soon dissipate. This kind of emotion regulation, reappraisal—changing how you think about a situation in order to diminish your emotional response—has long been regarded as an adaptive strategy for regulating one’s emotions [1,2]. Despite being an adaptive strategy, reappraisal may have cognitive costs: that is, thinking of a neutral interpretation of an event may be effortful, and your ability to execute the strategy effectively might also depend on your prior experience with reappraisal.

A useful framework for studying questions about the consequences of reappraisal is the process model of emotion regulation [3], which predicts that different emotion regulation strategies target different stages of the emotional response as it unfolds, with varying consequences for different components of emotion [1,4]. For example, reappraisal and distraction (focusing one’s attention elsewhere) are considered antecedent-focused strategies that are effective in reducing emotional experience because they have a chance to change the course of the emotional response as it develops. In contrast, suppression (maintaining a neutral facial expression in the face of an emotionally evocative event) is considered a response-focused strategy that is engaged only after the emotional response has fully developed. As such, suppression is less effective in changing the emotional experience. In a recent revision of this model, it was proposed that different strategies target different attentional processes. As a result, different strategies require different amounts of effort and have different consequences for the emotional response [5]. This perspective is known as the process-specific timing hypothesis. An early selection process (targeted when using distraction) involves directing selective attention to one or another perceptual input and is relatively effortless and therefore more likely to be effective. In contrast, the late selection process (targeted when using reappraisal) involves competition between different semantic representations. In the case of reappraisal, there is competition between an emotional representation—someone might be terribly hurt in a car accident—and a more neutral representation—they are close to a hospital and will probably be fine. Conflict between semantic representations at the late selection stage of processing places more demands on cognitive resources and will be less effective. Behavioural (e.g., performance on a measure of self-control after engaging in reappraisal) and self-report data (e.g., ratings of sadness in response to a sad movie) support this view [6,7].

The process-specific timing hypothesis gives rise to some key predictions about the effects of reappraisal. One prediction is that strategies targeting the late selection stage are more sensitive to the effects of stimulus intensity than strategies occurring at the early selection stage. That is, strategies targeting late selection should be more effortful and less effective [5]. In support of this assertion, individuals are less likely to choose reappraisal than distraction to regulate their emotions when stimuli are more intense, based either on subjective ratings of intensity or on neural measures of intensity, such as the late positive potential [8,9]. Also, reappraisal is effective for reducing emotional feelings when initiated early, but not late, during a sad film [10]. Furthermore, initiating reappraisal, but not distraction, late during a film, is associated with subsequent decrements in self-control on a Stroop task, suggesting a depletion of cognitive resources after reappraisal when intensity is high [6]. Thus, reappraisal choice, efficacy, and effort are all influenced by emotional intensity. In both these latter studies, stimulus intensity was manipulated indirectly by delaying reappraisal initiation—i.e., stimulus intensity was assumed to increase over the course of the film stimuli. Physiological data support this assumption, with participants demonstrating increased heart rate deceleration, facial muscle activity, and startle response over the course of viewing an emotional film or emotional pictures [11,12]. However, variables other than stimulus intensity might also change as people are exposed to the unfolding story in the emotional film for longer. For example, semantic representations may become more elaborated over the course of viewing an emotional film, perhaps making it more difficult to generate alternative appraisals. To eliminate such confounds, it may be beneficial to manipulate stimulus intensity directly, rather than through manipulating the timing of the reappraisal response.

An alternative approach to test the effects of stimulus intensity on reappraisal effort is to use stimuli that are already known to vary in their intensity. However, there is a lack of research taking this approach. In the current experiment, we sought to examine how intensity influences the cognitive effort of reappraising pleasant and unpleasant stimuli. We included pleasant stimuli because most research on the cognitive costs of emotion regulation has focused on unpleasant stimuli (for an exception, see [13]), yet people do sometimes regulate their emotions in contra-hedonic ways—in other words, by down-regulating pleasant feelings [14,15]. Given that people do down-regulate positive emotions, it is important to examine this phenomenon more closely to determine whether the processes and outcomes are the same as for the down-regulation of negative emotions. Some studies have compared the subjective consequences of emotion regulation of unpleasant and pleasant stimuli and have found that people can effectively down-regulate their emotions during both pleasant and unpleasant film clips, using reappraisal [16]. Furthermore, in an experience sampling study, Riediger, Wrzus, Schmiedek, Wagner, and Lindenberger [17] found that working memory performance was lower when people were engaged in down-regulation of positive emotions than in down-regulation of negative emotions, even when controlling for current positive and negative affect. This finding suggests that it is more cognitively costly to regulate one’s emotions to positive than negative stimuli. However, the specific emotion regulation strategies employed were not measured in that study. To our knowledge, no published studies have directly compared the cognitive costs of reappraisal for pleasant and unpleasant stimuli in a controlled laboratory environment. As an initial exploration of this issue, we sought to directly compare costs of reappraising emotions to pleasant and unpleasant stimuli that were matched in intensity based on normative ratings of arousal.

A second key prediction arising from the process-specific timing view is that processing at the late selection stage can be facilitated by experience. That is, training and practice in reappraisal, which involves competing representations at the late selection stage, should reduce the cognitive costs of implementing reappraisal and increase success [5]. People who report using reappraisal more in everyday life (habitual reappraisal) show greater decreases in negative feelings when reappraising negative pictures in the laboratory [18]. In addition, exposing participants to four sessions of reappraisal training increased their ability to reduce their negative feelings when viewing negative pictures as well as their experience of stress in their lives [19]. Other studies have found no association between self-reported use of reappraisal in everyday life and reappraisal ability as measured in laboratory tasks [20,21]. However, we have been unable to find research directly assessing how the cognitive costs of reappraisal vary for individuals with more or less frequent use of reappraisal. That is, do individuals with greater use of habitual reappraisal exert less effort to reappraise than individuals with less? We sought to examine whether reappraisal effort depended on prior reappraisal experience.

The current study was therefore designed to test these two key predictions of the process-specific timing view: that high intensity stimuli are more cognitively costly to reappraise than low intensity photos, and that greater habitual reappraisal predicts lower cognitive costs of reappraisal. To test these predictions, we used a dual-task paradigm that has previously been shown to be sensitive to the cognitive costs of reappraisal [22]. Participants watched pleasant and unpleasant picture stimuli that varied in intensity, and simultaneously performed a simple reaction time task. On each trial, they either reappraised, suppressed their emotions, or viewed the picture with instructions not to control their emotions. Suppression was included because we have used suppression as a comparison strategy in previous studies using this dual-task paradigm and wished to replicate those findings [22]. Furthermore, suppression has been widely studied in the emotion regulation literature and is considered a less adaptive form of emotion regulation, more cognitively costly, and does not produce reliable changes in emotion experience [22,23]. However, our particular focus in the current study was the comparison between reappraisal and view. We considered adding distraction as a comparison condition. However, when we conducted pilot tests in the laboratory with four emotion regulation conditions in a similar task, participants reported that they were fatigued and becoming desensitised to the images. As such, we decided to keep to three conditions, to ensure that participants could continue to put in their best effort on the emotion regulation and reaction time tasks for the duration of the experiment.

Prior research has shown that reappraisal results in slower reaction times than viewing unpleasant stimuli with no regulation instructions [22]. Based on the process-specific timing view, we predicted that the slowing in reaction times due to reappraisal would be greater for high than low intensity unpleasant pictures. We also tested whether this prediction would hold for pleasant stimuli. Furthermore, we predicted that greater self-reported use of reappraisal in everyday life (habitual reappraisal) would be associated with lower subjective emotional intensity and smaller decrements in reaction time performance. For suppression, we expected to replicate our previous findings of minimal cognitive costs of suppression without changes in subjective experience [22].

## Method

### Participants

The experiment received approval from Thompson Rivers University’s Research Ethics Board. All participants gave written informed consent. One hundred first year undergraduate students from a small university took part in the study. In a prior study examining the cognitive costs of reappraisal with the same paradigm [22], there was an effect size of *d* = .72. Based solely on this value, a study would need 23 participants to achieve .95 power to replicate those effects, using a within-subjects design. However, given the addition of other predictors in the experiment (namely stimulus intensity and valence) and that we would be testing for potential three-way interactions (between emotion regulation strategy, stimulus intensity, and stimulus valence), we aimed for a conservative sample size of 100 for the study.

Participants reporting recent diagnosis or treatment for any mental disorder, blood/injury/injection phobia, pregnancy, or cardiovascular or pulmonary conditions were not eligible to take part. A manipulation check, where participants indicated how they had implemented each of the emotion regulation strategies, indicated that ten participants had not correctly followed instructions (e.g., reported using suppression during reappraisal trials), and two participants started, but did not complete the study. As such, those participants’ data were not included in the analyses (final *N* = 88, 55 females, 33 males).

### Procedure and materials

The experimenter tested participants individually in sessions lasting approximately 40 minutes. Participants completed the following measures, only: the emotion regulation task and a measure of habitual reappraisal (the Emotion Regulation Questionnaire, described below). The design was within-subjects, with participants reappraising, suppressing, and viewing pictures that were either pleasant or unpleasant.

#### Emotion regulation task

Following similar procedures to previous research [22], participants watched a total of 96 images from the International Affective Picture System [24], presented using E-Prime experiment software. Forty-eight pleasant (e.g., paraglider) and 48 unpleasant (e.g., plane crash) pictures were selected that varied in their normative arousal ratings to include images both high and low in arousal. Pleasant and unpleasant stimuli were matched for arousal and valence (absolute deviation from the neutral mid-point of 5 on the rating scale of 1 to 9). IAPS picture numbers were as follows: pleasant, high arousal, 8170, 9156, 7405, 2300, 5470, 1650, 8163, 5626, 5621, 8034, 1710, 8200, 2045, 8370, 4597, 8030, 8499, 2345, 8080, 8400, 8116, 8490, 1811, 4626 (valence M = 7.35, SD = 0.45, arousal M = 6.15, SD = 0.54); pleasant, low arousal, 7284, 5201, 8320, 7530, 8032, 8465, 2060, 5820, 2037, 7472, 2302, 2387, 2501, 2791, 5991, 5551, 4536, 1333, 5635, 7238, 8050, 2151, 4622, 5220 (valence M = 6.62, SD = 0.46, arousal M = 3.91, SD = 0.38); unpleasant, high arousal, 9620, 9611, 7380, 6250, 9800, 3019, 3213, 6570, 9810, 9910, 8485, 6830, 2981, 6821, 6831, 9300, 2730, 6220, 9414, 3550, 2683, 9904, 9181, 3220 (valence M = 2.52, SD = 0.31, arousal M = 6.14, SD = 0.38); unpleasant, low arousal, 9291, 6010, 7078, 7079, 7521, 2490, 9045, 9471, 2039, 2399, 9190, 2455, 2590, 7023, 9110, 9090, 9331, 2525, 2312, 2491, 2718, 9046, 9220, 9390 (valence M = 3.52, SD = 0.47, arousal M = 4.05, SD = 0.30).

Participants initially received detailed instructions in how to reappraise, suppress, and view, adapting procedures from Richards and Gross (Study 2) [23]. For reappraisal, participants were given the option to generate a reinterpretation of the stimulus or to view it in a detached way (cf. [25]). Suppression involved concealing facial expressions of emotion [26]. During training participants responded to the reappraisal trials aloud, and received feedback and coaching from the experimenter to ensure correct application of each strategy. The instructions emphasised applying the strategy as instructed and avoiding the use of distraction or looking away.

Participants then received training on and completed ten practice trials of the reaction time task. In this task, participants heard a 500 ms duration low (520 Hz) or high (1000 Hz) tone, occurring at 1000 ms after picture onset. Participants pressed one of two buttons on a response box to indicate whether the tone was high or low. Speed and accuracy were emphasised. Participants were required to achieve at least 80% accuracy with a mean reaction time of less than 1500 ms before proceeding with the task. All participants included in the final study met these criteria.

For the full task, participants watched images in four blocks of eight trials for each strategy (view, reappraise, or suppress), for a total of 12 blocks. Each block of eight trials included two of each stimulus type (pleasant, high arousal; pleasant, low arousal; unpleasant, high arousal; unpleasant, low arousal). Blocks were presented in quasi-random order, such that each three consecutive blocks contained one of each of strategy, with the constraint that the same strategy was never repeated on two consecutive blocks. Within blocks, the order of image presentation was quasi-random, such that the first four trials consisted of one of each image type in random order, and the last four trials also consisted of one of each image type in random order. We used blocks to minimise the likelihood of carry-over effects and any strategy switching cost on each trial. At the beginning of each block, the participant saw a short reminder of the strategy they were due to implement (Reappraise: “Change how you think about each picture so that you do not feel anything at all.” Suppress: “Try not to make any facial expressions at all.” View: “Please view each picture as you normally would.”). Prior to each trial, participants saw the instruction to “Reappraise,” “Suppress,” or “View,” for 1500 ms. Subsequently, there was a fixation cross for 1000 ms, followed by a 3500 ms picture presentation. The high or low tone occurred 1000 ms after picture onset. The tone was presented early after picture onset in order to shorten the length of the task overall and because pilot tests demonstrated costs of reappraisal as early as one second after picture onset. Furthermore, EEG research has shown that the late positive potential, reflecting processing of arousing features of emotional stimuli, can be attenuated by reappraisal as early as 1500ms after picture onset [27], suggesting that at least some change in processing to re-evaluate the stimulus may have started prior to this timepoint. To assess subjective negative affect, participants rated intensity and pleasantness of their feelings immediately after each picture offset. Intensity was introduced as, “How strong or intense your feelings are” (1 = not at all intense, 9 = extremely intense) and pleasantness as, “How pleasant or unpleasant the photo made you feel” (1 = extremely unpleasant, 9 = extremely pleasant). Although the self-report ratings alone were not of primary interest in the current study we included them in order to assess whether participants had successfully regulated their emotions (at least subjectively).

#### Emotion regulation questionnaire (ERQ)

The ERQ [28] consists of ten items assessing the use of reappraisal (e.g., “When I want to feel less negative emotion, I change the way I’m thinking about the situation”) and suppression (e.g., “I control my emotions by not expressing them”) in everyday life. Participants respond to each item on a scale from 1 (strongly disagree) to 7 (strongly agree). The scale yields a mean score for reappraisal and a mean score for suppression. The ERQ has good internal consistency and is a valid predictor of well-being and successful social relationships [28]. Participants completed the ERQ after the emotion regulation task to reduce the likelihood that contemplating their everyday use of reappraisal and suppression might influence how they implement them during the task.

#### Manipulation check

Finally, participants described and gave an example of how they responded on reappraisal, suppress, and view trials. We discarded all data from participants who reported using strategies that did not match the instructed strategies for each trial type.

### Data analysis

To manage the concern that participants with a large number of inaccurate or missing trials were not following instructions, we used a rule of thumb requiring 90% accuracy on the reaction time task for inclusion in the analyses. Reaction time data from five participants with more than 10% incorrect or missed trials were not included in the analyses. Mean accuracy for the remaining participants was 96% and so accuracy was not analysed further and all trials were included in the analyses. Responses faster than 200 ms were deleted. Reaction time data were positively skewed. Log reaction times resulted in normal distributions. The analyses of reaction time data were conducted both with raw reaction times and with log reaction times, yielding the same results. Therefore, raw reaction time analyses are reported here to facilitate interpretation. Raw reaction times in ms were converted to reaction times in s to give more manageable numbers in the final analyses. For the subjective ratings, one participant reported “9” for all intensity ratings (i.e., extremely intense) and “9” for all pleasantness ratings (extremely pleasant), regardless of photo valence, suggesting that they were not following instructions. Therefore, the subjective data (but not reaction time data) from that participant were excluded from the analyses. Another participant reported after completing the experiment that they had misinterpreted the pleasantness rating scale and responded backwards (i.e., 1 for pleasant and 9 for unpleasant, rather than vice versa) on about half the task. Data from that participant were excluded from the analyses of pleasantness ratings. An alternative approach to dealing with inaccurate reaction time responses and missing data is to exclude all inaccurate trials from analyses and to exclude non-compliant participants from all analyses. Analyses conducted in this fashion resulted in the same pattern of significant effects (main effects and interactions), except in one case, where the interaction between strategy and arousal in their effects on pleasantness ratings was no longer significant.

Data were analysed with SPSS version 21, using the mixed models analysis of variance procedure. We chose this approach for three reasons, following recommendations in the literature on mixed models [29,30]: 1) To account for the fact that individual photos varied in intensity and valence. Rather than computing mean scores for each condition, each trial is included in the analyses, with intensity and valence of the photos as covariates. 2) To account for within-subject variability by including participant as a random effect. 3) To include stimulus as a random effect to account for variations in responses across stimuli that were not related to stimulus intensity or valence.

We used restricted maximum likelihood (REML) estimation for the final models and a variance components covariance matrix structure for the random effects. Maximum likelihood (ML) estimation was used when comparing model fit for nested models varying in the fixed effects included. The goal was to achieve parsimonious final models that included all significant fixed effects and hypothesis-driven interactions.

For the reaction time analyses, we used a top-down approach, starting with a full model with strategy (reappraise, suppress, or view), arousal (normative ratings from the IAPS included as a covariate), and valence (normative ratings from the IAPS included as a covariate), and their two- and three-way interactions as fixed predictors. View was entered as the reference category. Normative arousal and valence were included as continuous covariates in order to better account for the variability in these dimensions and because they may influence responses. Gender was initially included in the model as a main effect only. Although gender has been shown to influence neural activity in response to emotional stimuli [25], this was not the focus of the current study, and gender differences in neural activity during emotion regulation are not thought to reflect differing levels of effort for men and women [31,32]. However, we wished to account for the finding that overall, men and women respond differentially to emotional stimuli [33,34]. Notably, the results were the same and model fit improved when gender was not included in the model. Therefore, for simplicity, the results are provided without gender in the analyses.

The same approach was used to assess the effects of strategy, arousal, and valence on subjective ratings of intensity and pleasantness. For both pleasantness and intensity ratings, the best model fit was obtained without gender and without the interaction between strategy, valence, and arousal, so gender and the three-way interaction terms were omitted from the final models.

To examine how habitual reappraisal influences the cognitive costs of emotion regulation (reaction times) and changes in subjective experience (intensity ratings only), we started with the final models from the analyses described above. In each case, we first added ERQ-Reappraisal and ERQ-Suppression as fixed effects. Then we added their interactions with strategy. We removed ERQ-Suppression and its interaction with Strategy as these were not significant predictors and resulted in poorer model fit. Adding interactions between the ERQ and arousal and valence resulted in a less parsimonious and less interpretable model with poorer fit.

Alpha was maintained at .05 throughout the analyses. The Sidak correction was used for any multiple comparisons (i.e., when examining differences between reappraise and view as well as between suppress and view). At present, there are no widely-agreed upon methods for calculating standardized effect sizes for mixed models analyses. In addition, as argued by Baguley [35], simple (unstandardized) effect sizes, when accompanied by confidence intervals to indicate the range of possible values for the effect, have the benefit of being uninfluenced by the reliability of the measures, restriction of the range, and differences in study design and of being more interpretable, since they are given in the original units of measurement. Therefore, we have reported point estimates of differences between means—subjective ratings of pleasantness or intensity on the 9-point scale or reaction times in milliseconds—with 95% confidence intervals.

## Results

### Subjective ratings of affect as a function of strategy, arousal, and valence

Analyses of subjective ratings are presented first in order to confirm that participants had successfully regulated their emotions, at least based on self-report. For pleasantness ratings, there was no significant three-way interaction between strategy, arousal, and valence, so the three-way interaction was excluded from the final model. Participants reported pleasant stimuli to be more pleasant than unpleasant stimuli, *F*(1, 92.0) = 17.78, *p* = .001, and low arousal stimuli to be more pleasant than high arousal stimuli, *F*(1, 92.0) = 18.53, *p* = .001. However, the effect of valence was moderated by strategy, *F*(2, 8101.9) = 118.44, *p* = .001. Specifically, for both reappraisal and suppression, compared to the view condition, pleasantness ratings decreased (came closer to 5 = neutral) for pleasant images and increased (came closer to 5 = neutral) for unpleasant images, *b* = -.25, *t*(8097.8) = -15.35 *p* = .001, 95% CI [-.278, -.216], for reappraisal versus view, and *b* = -.11, *t*(8102.4) = -6.64, *p* = .001, 95% CI [-.139, -.076], for suppress versus view.

**Fig 1:**
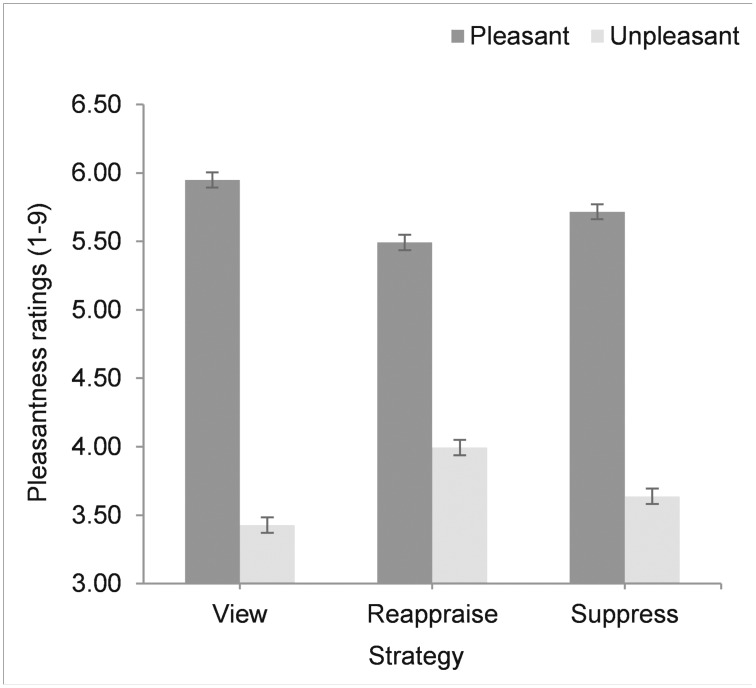
Mean pleasantness ratings for pleasant and unpleasant pictures (one standard deviation above and below the mean), for each emotion regulation strategy. Error bars represent standard errors after conducting spotlight analyses.

In order to examine the effect of strategy at different valences, we conducted spotlight analyses, examining differences in pleasantness ratings between view, reappraise, and suppress for high and low valence stimuli. Valence was set at +1.0 (pleasant) and -1.0 (unpleasant) standard deviations from the mean (we did not examine mean values of valence, which would have been for neutral pictures, not included in the study). We applied the Sidak correction for multiple comparisons. For pleasant pictures, pleasantness ratings were closer to neutral for suppress than view, *M* = .232, *p* = .001, 95% CI [.120, .345], and for reappraise than suppress, *M* = .224, *p* = .001, 95% CI [.337, .111]. For unpleasant pictures, the direction of effects was the same: pleasantness ratings were closer to neutral for suppress than view, *M* = .212, *p* = .001, 95% CI [.099, .325], and for reappraise than suppress, *M* = .356, *p* = .001, 95% CI [.243, .469]. In other words, both reappraisal and suppression led to more neutral feelings (less pleasant or unpleasant, according to the stimulus valence) compared to view (Fig 1).

Interactions indicated that the difference in pleasantness ratings between low and high arousal stimuli was greater for negative than positive photos, *F*(1, 92.0) = 7.99, *p* = .006, *b* = .036, *t*(92.0) = 2.83, *p* = .006, 95% CI [.011, .062]. In addition, there were increases in pleasantness ratings (ratings moving closer to neutral) from view to reappraise for high, but not low arousal pictures, *F*(2, 8104.2) = 3.08, *p* = .046. However, interpretation of this interaction is not that meaningful because it collapses across pleasant and unpleasant photos, for which pleasantness ratings were expected to decrease and increase, respectively. Therefore, we focused the remainder of our analyses of subjective data on the intensity ratings.

**Fig 2:**
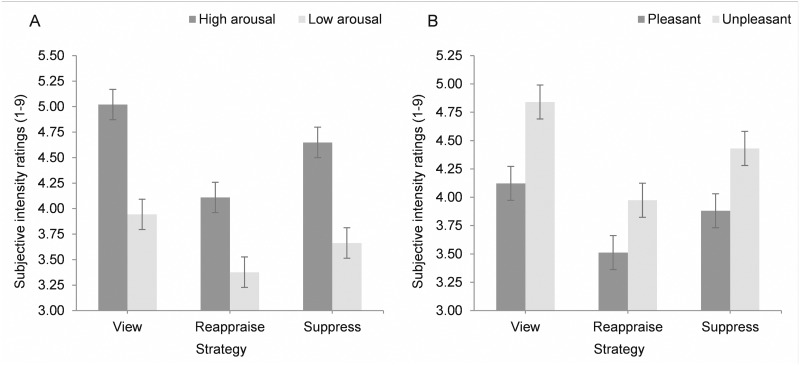
Mean subjective intensity ratings for high and low arousal (one standard deviation above and below the mean) pictures (A) and pleasant and unpleasant (one standard deviation above and below the mean) (B) pictures, for each emotion regulation strategy. Error bars represent standard errors after conducting spotlight analyses.

For subjective intensity ratings, there was no significant three-way interaction between strategy, arousal, and valence, so the three-way interaction was excluded from the final model. Participants reported higher subjective intensity ratings for high than low arousal photos and for unpleasant than pleasant photos, *F*(1, 92.0) = 70.75, *p* = .001, *F*(1, 92.0) = 5.35, *p* = .023. There were also significant interactions between strategy and arousal, *F*(2, 8189.9) = 11.08, *p* = .001, between arousal and valence, *F*(1, 92.0) = 18.30, *p* = .001, and between strategy and valence, *F*(2, 8190.7) = 5.98, *p* = .003. Specifically, the difference in intensity ratings between view and reappraise was greater for high than low arousal photos, *b* = -.149, *t*(8184.2) = -4.54, *p* = .001, 95% CI [-.214, -.085] and the difference in intensity ratings between view and reappraise was greater for unpleasant than pleasant photos, *b* = .062, *t*(8186.8) = 3.40, *p* = .001, 95% CI [.026, .098]. Similarly, the difference in intensity ratings between view and suppress was greater for unpleasant than pleasant photos, *b* = 0.041, *t*(8190.4) = 2.23, *p* = .026, 95% CI [.005, .076]. In sum, both reappraisal and suppression were more effective in reducing intensity ratings (compared to view) for unpleasant than pleasant pictures and for high than low arousal photos (Fig 2).

### Reaction times as a function of strategy, arousal, and valence

**Fig 3:**
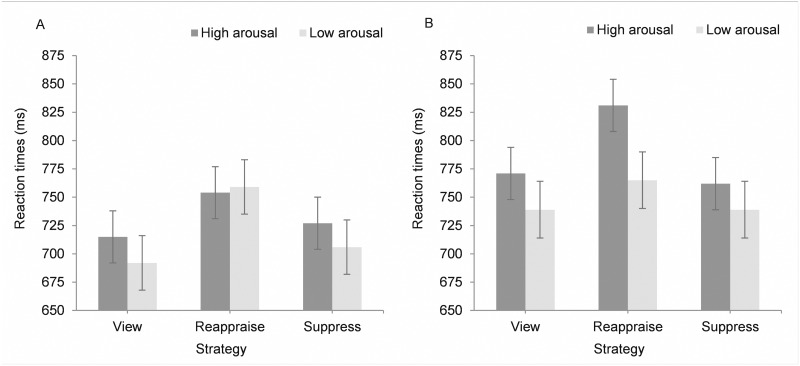
Mean reaction times during pleasant (A) and unpleasant (B) pictures for high and low arousal (one standard deviation above and below the mean) pictures, for each emotion regulation strategy. Error bars represent standard errors after conducting spotlight analyses.

Reaction times were slower during high than low arousal stimuli, *F*(1, 91.8) = 5.76, *p* = .018. There was also a significant three-way interaction between strategy, arousal, and valence, *F*(2, 7700.0) = 3.22, *p* = .040. No other main effects or interactions were significant, all *p*’s > .05. The three-way interaction was accounted for by an interaction between arousal and valence for reappraisal compared to the view condition, *b* = -.0057, *t*(7700.0) = -1.98, *p* = .047, 95% CI [-.011, -.00006]. Specifically, for unpleasant but not pleasant pictures, the high arousal photos resulted in greater slowing of reaction times for reappraise compared to view than the low arousal photos. That is, as predicted, the cognitive costs of reappraising were greater for high arousal, unpleasant stimuli than low arousal, unpleasant stimuli. We conducted spotlight analysis to examine the cognitive costs of reappraising (difference in reaction times between reappraise and view) for high and low arousal photos that were pleasant and unpleasant (with valence and arousal set at either -1.0 or +1.0 standard deviations from the mean). For high arousal stimuli, reappraisal of both unpleasant and pleasant pictures resulted in reaction time costs, *M* = 58 ms, *p* = .001, 95% CI [29, 86], and *M* = 40 ms, *p* = .002, 95% CI [12, 68], respectively, whereas for low arousal stimuli, only reappraisal of pleasant pictures resulted in reaction time costs, *M* = 68 ms, *p* < .001, 95% CI [35, 100] (applying the Sidak correction) (Fig 3).

### Role of everyday life reappraisal in predicting affect

**Fig 4:**
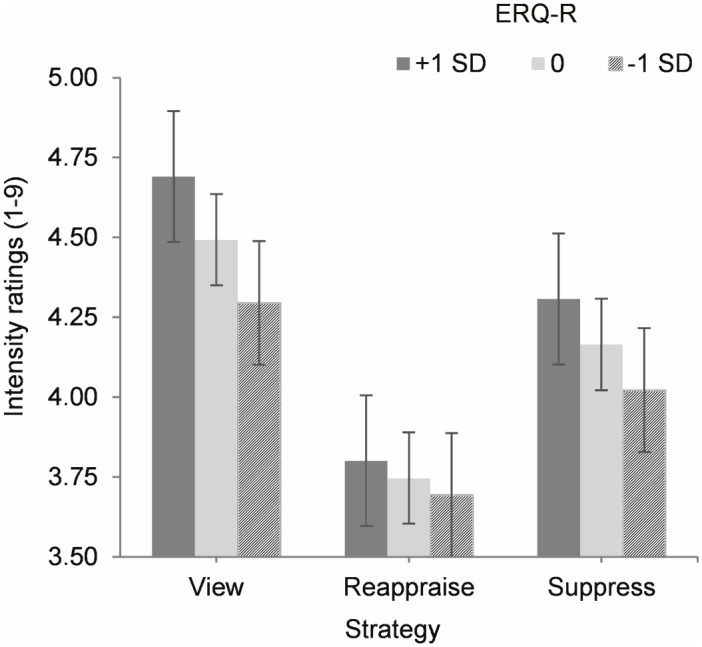
Intensity ratings for participants with varying habitual reappraisal (one standard deviation above, at, or one standard deviation below the mean on the Emotion Regulation Questionnaire-Reappraisal scale (ERQ-R)), according to emotion regulation strategy. Error bars represent standard errors after conducting spotlight analyses.

To test whether habitual reappraisal would be associated with more effective reappraisal (at least subjectively), we explored the role of ERQ-Reappraisal scores in predicting intensity ratings. ERQ-Reappraisal scores alone did not predict intensity ratings, *F*(1, 88.0) = .93, *n*.*s*. However, the interaction between ERQ-Reappraisal and strategy was significant, *F*(2, 8196.9) = 7.35, *p* = .001. Participants who used reappraisal more in everyday life also reported greater reductions in intensity ratings between reappraise and view, *b* = -.149, *t*(8203.2) = -3.80, *p* = .001, 95% CI [-.226, -.072]. Therefore, as predicted, those with more habitual reappraisal use were more effective at reducing their subjective feelings of emotion than those with less habitual reappraisal use. Spotlight analysis with ERQ-Reappraisal scores at -1.0, 0.0, and +1.0 standard deviations from the mean indicated that individuals at all levels of ERQ-Reappraisal experienced significant reductions in subjective intensity ratings when reappraising compared to when viewing the stimuli, all *p*’s = .001 (Fig 4).

### Role of everyday life reappraisal in predicting reaction times

**Fig 5:**
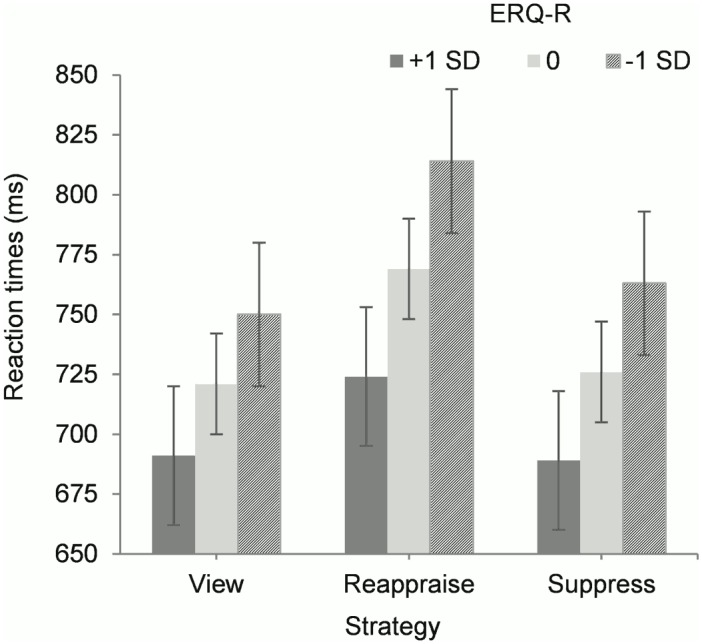
Mean reaction times (ms) for participants with varying habitual reappraisal (one standard deviation above, at, or one standard deviation below the mean on the Emotion Regulation Questionnaire-Reappraisal scale (ERQ-R)), according to emotion regulation strategy. Error bars represent standard errors after conducting spotlight analyses.

When ERQ-Reappraisal scores and their interaction with strategy were included in the model, the interaction between strategy, arousal, and valence in predicting reaction times was still significant, *F*(2, 7698.3) = 3.23, *p* = .039. Alone, ERQ-Reappraisal was a marginally significant predictor of reaction times, *F*(1, 83.4) = 3.79, *p* = .055. More importantly, there was a significant interaction between strategy and ERQ-Reappraisal, *F*(2, 7706.4) = 3.03, *p* = .048. The interaction was accounted for by smaller increases in reaction times between view and reappraise (suggestive of less effort) for participants reporting greater use of reappraisal in everyday life, *b* = -.016, *t* = -.246, *p* = .014, 95% CI [-.029, -.003]. Spotlight analyses with ERQ-Reappraisal scores at -1.0, 0.0, and +1.0 standard deviations from the mean indicated that individuals at all levels of ERQ-Reappraisal experienced significant increases in reaction times from view to reappraise, all *p*’s = .001 (Fig 5).

## Discussion

The current study provides a direct demonstration of the variation in cognitive costs of reappraisal according to emotional stimulus intensity and individual frequency use of reappraisal in everyday life. According to the process-specific timing hypothesis, reappraisal targets the late processing stage of attention, where semantic mental representations compete for processing [5]. During reappraisal, conflict arises between two representations—the initial, emotional, appraisal of the stimulus and the more neutral reappraisal. Two key predictions derived from this model are that: the cognitive costs involved in reappraisal depend on stimulus intensity, with higher intensity stimuli resulting in higher conflict and therefore greater cognitive costs, and that individuals who use reappraisal more frequently experience lower cognitive costs. In the current study, we tested the first prediction by directly manipulating stimulus intensity and observing the effects on concurrent reaction time performance while participants followed instructions to regulate or not regulate their emotional experience. The difference in reaction times between regulation and no regulation conditions gives an indication of cognitive costs. In addition, because prior research has typically neglected to make direct comparisons of the cognitive costs of reappraising positively- and negatively-valenced stimuli, we included both stimulus types in the experiment. To test the second prediction, participants also reported on their use of reappraisal in everyday life, and we assessed the relation between reappraisal scores, the cognitive costs of reappraising, and subjective success of reappraisal.

In support of the first prediction, we found that reappraisal was more cognitively costly for high than low arousal stimuli, with the costs being eliminated for low intensity images. However, this effect was observed for unpleasant photos only. The finding complements existing research showing that delaying the initiation of reappraisal of negative stimuli also increases its costs and reduces its effectiveness [6,10]. For example, Sheppes and Meiran [6] found that when people began to reappraise a sad film half-way through (rather than at the beginning of) a sad film, they showed greater conflict on a Stroop task relative to a distraction condition. In another study, initiating reappraisal late during a negative film—when negative feelings are presumably more intense—resulted in greater negative feelings than initiating reappraisal prior to the onset of or early during the negative film [10]. In both these aforementioned experiments, stimulus intensity was manipulated indirectly, by varying the time at which participants initiated reappraisal. Importantly, in the current study, we directly manipulated stimulus intensity rather than relying on reappraisal timing as a proxy for intensity. In other words, whether stimulus intensity is manipulated through delaying the onset of reappraisal (as in prior research) or through using stimuli of different intensities (as in the present study), increasing the intensity results in increasing cognitive costs. This result therefore supports the process-specific timing hypothesis, which would suggest that high intensity stimuli are more costly to reappraise because there is greater conflict between the emotional and neutral (reappraised) semantic representations of the emotional event [5].

The effect for pleasant photos was different. For pleasant photos, reappraisal of both high and low arousal photos resulted in similar cognitive costs. One possible explanation, which should be tested in future research, is that low intensity positive pictures are relatively difficult to reinterpret in a more neutral way, because they already appear to be fairly close to neutral (e.g., a scene of a footbridge over a river, people walking on a beach). In this case, the costs may arise not because the two semantic representations of the photo (the original representation and the reappraisal) are so different, but because more effort is required to generate a reappraisal that is different from the original perspective on the photo. In other words, the costs of reappraisal may not just happen at the late attentional selection stage, but also just in the course of generating a reappraisal (cf. [36]). Further research could provide a more nuanced view of how both pleasant and unpleasant stimuli are reappraised, and how the cognitive costs of reappraisal vary across the timecourse of an emotional response.

In support of the second prediction, participants reporting more habitual use of reappraisal demonstrated lower cognitive costs of reappraising compared to participants reporting less habitual use of reappraisal, yet were still able to reap the subjective benefits. Indeed, there were greater reductions of negative affect in participants with more reappraisal experience. Although this finding was correlational (habitual reappraisal was based on self-reports of use of reappraisal in daily life), it is congruent with recent experimental work demonstrating that reappraisal training in the laboratory increases the effectiveness of reappraisal for reducing subjective negative affect in response to aversive photos [19], and with work showing that people who report using reappraisal more in everyday life show greater decreases in negative feelings when reappraising negative pictures in the laboratory [18]. The current finding adds to the subjective report data by demonstrating the variation in cognitive costs of reappraisal for individuals with different frequencies of reappraisal use. Although it has yet to be demonstrated that reappraisal training results in the same cognitive processes being targeted as in individuals who already use reappraisal habitually in everyday life, the findings do provide converging evidence that the costs of reappraisal can be mitigated through experience and practice. Again, the finding also provides further support for the process-specific timing hypothesis, which predicts that processing at the late selection stage can be facilitated by experience. Furthermore, the results suggest that there may be some automatization of reappraisal in individuals with a greater frequency of reappraisal use in every day life. Further studies could explore this interpretation further.

The findings for suppression were different. In fact, for suppression, we found no cognitive costs. Previous research has found that suppression impairs incidental memory for information presented during stimulus viewing [26], but does not reliably slow reaction times [22]. Perhaps the reaction time task used both in previous work [22] and in the current experiment is simply not sensitive to the costs of suppression. This may be in part because of the timing of the concurrent task: in the current study, participants responded on the reaction time task early during photo presentation. Suppression is considered a response-focused strategy that is implemented after the emotional response has unfolded, and so the costs of suppression may occur later—during or after stimulus presentation [22]. With respect to the effects of suppression on subjective feelings, we found that suppression reduced subjective feelings (both in terms of intensity and valence) compared to view, though the effects were smaller than for reappraisal (cf. [37]). Other studies have found no [23,26] or unreliable [22] effects of suppression, but these inconsistencies may be attributable to the different ways of measuring subjective emotions. For example, Richards and Gross [26] asked participants to rate the extent to which they felt a number of more discrete emotional states (e.g., distressed, upset, angry) after a set of slides. In the current experiment, participants rated their emotional experience using dimensions of valence and arousal after every photo. This latter approach may be more sensitive to small shifts in overall emotional experience, not specific to discrete emotions, on a trial by trial basis.

### Limitations and future directions

There are some limitations to the present study. Although we experimentally demonstrated the varying cognitive costs of reappraising unpleasant stimuli, we did not replicate this finding with pleasant stimuli. This seems contrary to the predictions of the process-specific timing hypothesis, where stimulus intensity (regardless of valence) is expected to predict reappraisal costs [5]. However, a few variables may account for this discrepancy.

First, reappraisal, with the goal of a less emotional, more neutral response, may be expected to draw on different cognitive processes according to whether the stimuli are unpleasant or pleasant. For example, reappraising unpleasant stimuli may fit with a hedonic goal, which, in the absence of competing instrumental goals, may be prioritised by participants. On the other hand, reappraising pleasant stimuli may conflict with such hedonic orientations, influencing the cognitive costs of down-regulating emotions to pleasant stimuli.

Second, although pleasant and unpleasant stimuli were matched a priori on ratings of arousal based on normative ratings, participants reported more intense emotional responses during unpleasant than pleasant photos (in the view condition), suggesting that the matching had not been completely effective for this particular sample. This issue should be considered when interpreting the variability in cognitive costs of reappraising high and low arousal pleasant and unpleasant stimuli. We found no effect of intensity on the costs of reappraising pleasant stimuli, but this may be explained by the lower intensity ratings, and therefore restriction of the range of intensity ratings, for pleasant pictures. Further research should carefully compare costs of reappraising pleasant photos across the full range of intensity.

Third, the process-specific timing hypothesis focuses on one aspect of reappraisal only—the conflict between competing semantic representations of the stimulus occurring at the late attentional selection stage. Others have argued that reappraisal may make demands on working memory, conflict monitoring, and goal maintenance processes, as the individual chooses and implements a reappraisal strategy and then maintains and updates it [36]. As such, reappraisal is not a one-off process, but consists of multiple processes that unfold over time. Each process may have different costs that may be tapped by different or overlapping measures. Thus, with a coarse measurement of “effort” or “cognitive costs” such as was employed in the current study (reaction times 1000 ms after picture onset) it is hard to discern which components of reappraisal are being assessed.

Fourth, pleasant and unpleasant photos, and also high and low arousal photos, differ not only in their valence and arousal, but also in the picture content (e.g., stimuli with social content involving happy or aggressive interactions between people, versus those showing images of injury or disease). Such differences could also influence the costs of reappraisal.

These limitations notwithstanding, the findings are consistent with other research supporting varying cognitive costs of reappraisal according to negative stimulus intensity. Further work in this area is needed to distinguish among the various processes involved in the reappraisal response and how these processes may differ when reappraising positive and negative stimuli.

A second key limitation of the current study was the correlational nature of our test of the second prediction. That is, we did not experimentally manipulate participants’ habitual reappraisal. Rather, we simply observed that self-reported habitual use of reappraisal was associated with lower cognitive costs and greater subjective benefits of reappraisal. The findings do mesh with past work showing that reappraisal training predicts reappraisal ability [18]. However, it is possible that a third variable, such as working memory, might predict both the frequency of use of reappraisal in everyday life and the subjective benefits and costs of reappraisal as measured in the laboratory. Indeed, other research has shown that working memory performance predicts reappraisal ability [18,38]. Future research should directly test whether reappraisal training reduces cognitive costs. Another potential avenue would be to explore whether reappraisal training results in similar forms of reappraisal as observed in those with greater prior reappraisal experience.

Another limitation is that completing the reaction time task during emotion regulation serves as a distraction from either the stimuli themselves or from the task of regulation. However, if this were the case, one would expect the task to be less sensitive to detecting the effects of stimulus characteristics and emotion regulation type on reaction time performance and subjective ratings. We were able to demonstrate the varying costs of reappraisal according to stimulus intensity in spite of the potentially distracting effects of the reaction time task. Of course, there remains the possibility that the tone task serves as a greater distraction on low intensity than high intensity trials, and this could account for the variability in reaction time performance across photos of different intensity. Future research should ensure that this explanation is ruled out.

A further limitation is that there were multiple ways in which participants could have reappraised the pictures on each trial. The reappraisal instructions were quite general and suggested at least two ways in which participants could reappraise—taking a detached perspective or generating an alternative interpretation of the stimulus. The manipulation checks indicated that different participants reported using each of these strategies. However, in the manipulation check we only asked participants to provide one example of a reappraisal rather than asking which reappraisal strategy was used on a trial by trial basis. Therefore, it is unknown how participants reappraised on each trial. Further research should examine the different types of reappraisal and their respective demands on cognitive resources.

It is also important to note that the process-specific timing hypothesis makes specific predictions about the costs and effectiveness of distraction under varying levels of stimulus intensity. Therefore, future research should include a comparison of distraction with reappraisal.

Another direction for future research would be to give participants a longer period of time to reappraise each image. In the current study, we used a short picture duration because pilot testing indicated that we can observe cognitive costs of reappraisal as early as one second after picture onset and research shows attenuation of the late positive potential as early as 1500ms after picture onset [27]. However, future work could examine further the time course of cognitive costs when participants are given longer to reappraise. Prior research has demonstrated that the cognitive costs of reappraisal diminish over time [22]. We would predict that this change would be slower for high than low intensity stimuli.

Finally, much research to date has explored the cognitive costs of emotion regulation using either film or photographic stimuli. However, little is known about how these costs may play out in emotionally evocative situations in everyday life. Would reappraisal of personally relevant emotional situations result in greater cognitive costs? For instance, what if you know that the ambulance that passes you has a loved one in it: would it be more cognitively effortful to employ reappraisal in this situation than if it was a stranger?

### Conclusions

In sum, the current research was designed to test two predictions of the process-specific timing hypothesis of emotion regulation: that the cognitive costs of reappraisal increase with increasing emotional intensity, and that habitual use of reappraisal reduces those cognitive costs. The study produced two novel findings in support of these predictions: the costs of reappraisal were greater for high than low intensity stimuli, though this only held for unpleasant, not pleasant, photos. In addition, participants with more habitual use of reappraisal experienced lower cognitive costs of reappraisal than participants with less habitual use of reappraisal, while still experiencing reduced subjective feelings of emotion. Importantly, we directly manipulated stimulus intensity to support the first hypothesis, and we measured cognitive costs (rather than reappraisal ability based only on subjective reports) to support the second hypothesis. Overall, the findings lend support to the key assumptions of the process-specific timing hypothesis, but more research is required to ascertain how the costs of reappraisal vary over time and for stimuli of different valence (pleasant and unpleasant).
